# Web alert: Two‐phase biocatalysis

**DOI:** 10.1111/1751-7915.14314

**Published:** 2023-07-24

**Authors:** Lawrence P. Wackett

**Affiliations:** ^1^ Department of Biochemistry, Molecular Biology & Biophysics, BioTechnology Institute University of Minnesota St. Paul Minnesota USA

Microfluidic droplets

https://www.ncbi.nlm.nih.gov/pmc/articles/PMC8616014/

This review article covers two‐phase biocatalysis in microfluidic droplets. This often serves to overcome mass transport issues that can limit enzyme catalysis with water‐insoluble compounds.

Two‐step organic synthesis biocatalysis

https://pubs.acs.org/doi/10.1021/acs.oprd.6b00232

This study used whole‐cell catalysts in a microaqueous phase within organic solvents to catalyse multiple enzymatic steps for making a pharmaceutical intermediate.

Flow biocatalysis

https://www.frontiersin.org/articles/10.3389/fctls.2023.1154452/full

This review covered new developments in flow biocatalysis during the years of 2020–2022.

Encapsulated *Pseudomonas* biotransformation

https://microbialcellfactories.biomedcentral.com/articles/10.1186/s12934‐023‐02073‐7

In this report, the authors describe optimization of (S)‐2‐hydroxypropiophenone synthesis by free and encapsulated cells of *P. putida*.

Biocatalysis methods

https://www.nature.com/articles/s43586‐021‐00044‐z

This is a broad and extensive review of biocatalysis that includes many aspects for using enzymes to synthesis commercial chemicals.

Pickering emulsions with yeast enzyme

https://pubs.acs.org/doi/10.1021/acssuschemeng.6b01776

This article describes the use of a small amount of solid particle emulsifier to make a Pickering emulsion system to catalyse reactions with *Candida antarctica* lipase B.

Pharma biocatalysis

https://www.almacgroup.com/knowledge/wp‐content/uploads/sites/10/2021/01/API_Practical‐Methods‐for‐Biocatalysis‐and‐Biotransformations‐2_Article‐2.pdf

This review chapter focuses on company‐based biocatalysis projects.

Microbial biocatalysis

https://www.mdpi.com/journal/catalysts/special_issues/microbes_biocatal

This links to a special issue of the journal “Catalysts” that includes examples of two‐phase microbial and enzyme biocatalysis.

Diene epoxidation by a *Pseudomonas*

https://www.sciencedirect.com/science/article/abs/pii/0141022986900761

This article highlights the production of 7,8‐epoxy‐1‐octene by non‐growing *Pseudomonas putida* PpG6 in two‐phase catalytic reaction.

